# Transmission networks of hepatitis C virus among HIV/HCV-coinfected patients in Guangdong, China

**DOI:** 10.1186/s12985-022-01849-4

**Published:** 2022-07-14

**Authors:** Xizi Deng, Zhiwei Liang, Weiping Cai, Feng Li, Junbin Li, Fengyu Hu, Yun Lan

**Affiliations:** Infectious Diseases Institute, Guangzhou Eighth People’s Hospital, Guangzhou Medical University, 8 Huaying Road, Baiyun District, Guangzhou, 510440 China

**Keywords:** Hepatitis C virus, Human immunodeficiency virus, Transmission network

## Abstract

**Background:**

Coinfection with hepatitis C virus (HCV) is common in human immunodeficiency virus (HIV)/acquired immunodeficiency syndrome (AIDS) patients due to shared routes of transmission. We aimed to investigate the characteristics of HCV subgenotypes among HIV/HCV-coinfected patients in Guangdong and explore the molecular transmission networks and related risk factors for HCV strains.

**Methods:**

Plasma samples were obtained from 356 HIV/HCV-coinfected patients for HCV *NS5B* region sequencing. A neighbor-joining phylogenetic tree was constructed to affirm HCV subgenotypes. The transmission networks based on maximum likelihood phylogenetic tree were determined by Cluster Picker, and visualized using Cytoscape 3.2.1.

**Results:**

A total of 302 HCV NS5B sequences were successfully amplified and sequenced from the 356 plasma samples. A neighbor-joining phylogenetic tree based on the 302 NS5B sequences revealed the profile of HCV subgenotypes circulating among HIV/HCV coinfection patients in Guangdong. Two predominant strains were found to be 6a (58.28%, 176/302) and 1b (18.54%, 56/302), followed by 3a (10.93%, 33/302), 3b (6.95%, 21/302), 1a (3.64%, 11/302), 2a (0.99%, 3/302) and 6n (0.66%, 2/302). A molecular transmission network of five major HCV genotypes was constructed, with a clustering rate of 44.04%. The clustering rates of subgenotypes 1a, 3a, 3b, 1b, and 6a were 18.18% (2/11), 42.42%, 52.38%, 48.21%, and 44.89%, respectively. Multivariate logistic regression analysis showed no significant effects from sex, age, transmission route, geographical region, baseline CD4 + T cell count or subgenotype (P > 0.05), except marital status. Married or cohabiting people (compared with unmarried people) had more difficulty forming transmission networks.

**Conclusions:**

In summary, this study, based on HCV NS5B subgenotypes, revealed the HCV subtype diversity and distribution among HIV/HCV-coinfected patients in Guangdong. Marital status inclined to be the factor influencing HCV transmission networks formation.

## Introduction

Hepatitis C virus (HCV) infection is a major cause of chronic liver diseases worldwide, such as cirrhosis, steatosis, and hepatocellular carcinoma [1]. HCV displays high levels of genetic diversity and has been differentiated into seven major genotypes and approximately 100 subgenotypes [2]. Different genotypes and subgenotypes differ in clinical outcomes, responses to treatment and epidemiology. Coinfection with HCV and human immunodeficiency virus (HIV) is common due to shared routes of transmission, including contaminated blood transfusion, sexual intercourse, and needle sharing in injection drug users (IDUs). HCV prevalence (HCV antibody positivity) was 0.60% among HIV-negative patients in China, while it was significantly higher among HIV-infected patients.Data from the China National Free Antiretroviral Treatment Program from 2010 to 2011 showed that 18.2% of 33,861 HIV-infected patients were co-infected with HCV [3]. Among HIV-infected patients in China, the overall prevalence of HCV was estimated to be 25.5–29.1%, with the highest rate of HCV co-infection among intravenous drug users and previous blood donors, exceeding 80% [4].HIV infection accelerates the natural progression of HCV infection; therefore, HCV coinfection has become the most common cause of death in HIV/ AIDS patients on antiretroviral therapy [5].

Viral sequence data such as that for HIV-1 can be used to reconstruct molecular transmission networks, approximating the transmission network and reflecting the transmission pathway of the virus between people [6]. Understanding the network through which the virus is transmitted is important for the successful implementation of treatment and prevention strategies [7–9]. The transmission network based on the HCV whole genome can better reflect the true transmission association. However, due to the diversity and secondary structure of HCV, it is difficult to obtain a large sample of whole-genome sequences in actual work. Clustering analyses of HCV genomes are generally performed using short sequences [10], and the nonstructural 5B viral region (*NS5B*) is considered an important target for HCV genotype and subgenotype identification [11–13] and has been applied to analyse transmission networks of HCV [14–17].

Phylogenetic analysis has been used successfully to identify and dissect HIV-1 transmission clusters. Understanding the structure and features of transmission clusters has the capacity to facilitate the identification of potential transmission partners and reveal the links between different populations and is important for the design of intervention programs [18]. In recent years, many molecular transmission networks have been reconstructed for HCV using the methodology previously developed for HIV sequence data [16]. Guangdong is one of the most developed provinces and has the largest population and the highest population density in China. The number of annually reported cases of hepatitis C in Guangdong has been increasing since 2005 [19]. In this study, we characterized the transmission patterns and influencing factors of molecular transmission networks for HCV among HIV/HCV-coinfected patients in Guangdong, China.

## Materials and methods

### Study population

Plasma samples for *NS5B* sequencing were obtained from 356 HIV/HCV-coinfected patients recruited between January 2010 and September 2013 from Guangzhou Eighth People’s Hospital. The inclusion criteria were as follows: (1) older than 18 years of age at time of enrollment, (2) positive HIV-1 ELISA (Beijing Wantai, China) with a confirmatory Western blot (MP Biomedicals, Singapore), (3) positive IgG or IgM anti-HCV ELISA (Zhongshan Bioengineering, China) and detectable HCV RNA > 1000 IU/ml (Guangzhou DAAN Gene Limited Company, China). The exclusion criteria were as follows:(1) positivity HBV surface antigen (HBsAg) ELISA (Zhongshan Bioengineering, China), (2) evidence of liver disease due to other etiology, (3) excessive alcohol consumption or using liver-toxic drugs, (4) previously received antiviral (HIV or HCV) treatment, and (5) individuals with decompensated cirrhosis and hepatocellular carcinoma (HCC), severe cytopenias, pregnancy, breast-feeding status, renal failure, heart failure, or an AIDS-defining illness. Demographic information, including sex, age, transmission route, marital status, geographical region, and baseline CD4 + T cell count, was obtained at patient enrolment and extracted through chart review.

### RNA extraction, amplification, and sequencing

Viral RNA was extracted from 140 µl of plasma using a QIAamp Viral RNA Mini Kit (Qiagen, Germany) following the manufacturer’s instructions. HCV *NS5B* (H77: 7996–8638 nt) fragments were amplified with a PrimeScript One-Step RT-PCR Kit and Premix Taq (Takara Bio, Dalian, China). The *NS5B* fragment was amplified with in-house degenerate primers (Table 1) under the following conditions: 95 °C for 3 min, followed by 35 cycles of 95 °C for 30 s, 55 °C for 40 s and 72 °C for 60 s for the first round and 95 °C for 2 min, followed by 35 cycles of 95 °C for 25 s, 55 °C for 40 s and 72 °C for 40 s for the second round. The PCR products were analysed using 1% agarose gel electrophoresis, and the positive products were sent for sequencing by a genomics company (Tianyi Huiyuan, China) with the primer R2.

**Table 1:** HCV primers for the *NS5B* region by genotype

Primers	Primer Sequences (5′-3′)	H77 location (nt)	Amplified length (bp)
*First round*			
Forward (F1)	CCACATCMRCTCCGTGTGG	7952–7970	696
Reverse (R1)	GGRGCDGARTACCTRGTCAT	8628–8647	
*Second round*			
Forward (F2)	ACMCCAATWSMCACBACCATCATG	7996–8018	643
Reverse (R2)	TACCTGGTCATAGCCTCCGTGAA	8616–8638	

### Identification of HCV subgenotypes

The reverse complements of the obtained sequences were determined and aligned by using BioEdit 7.0. Then, sequence alignments were performed with HCV subtyping references from the Los Alamos HCV Sequence Database (https://hcv.lanl.gov/). All sequences were manually edited. HCV subgenotypes were assigned based on phylogenetic analysis of *NS5B* region sequences. Neighbor-joining phylogenetic trees were constructed with the Kimura 2-parameter substitution model and evaluated by the bootstrap method with 1000 replicates by using MEGA 6.06.

### Analysis of HCV molecular transmission networks

The flow chart of transmission network analysis includes four steps [20]. First, PhyML 3.0 was used to construct a maximum likelihood phylogenetic tree (ML tree) using the GTR + G + I nucleotide substitution model. The phylogenetic tree’s reliability was determined with branch support based on the approximate likelihood ratio test (aLRT) with Shimodaira-Hasegawa (SH) supports of 1000 replicates [21]. Second, Cluster Picker [22] was used to determine extra transmission clusters with an intra-cluster maximum pairwise distance < 4.0% nucleotide substitutions per site [23] and bootstrap support value ≥ 0.9. Third, Mega 6.0.6 was used to calculate the Tamura-Nei 93 pairwise genetic distances to define the linkages within a cluster. Finally, the network data were visualized using Cytoscape 3.2.1 (http://cytoscape.org).

### Statistical analysis

The database was established in Excel, and the statistical analyses were performed using IBM SPSS V25.0 (SPSS Inc. Chicago, IL). Categorical variables were compared using Fisher’s exact tests. Univariate and multivariate logistic regression models were used to estimate the potential factors associated with transmission within clusters. The variables considered were sex, age, transmission route, marital status, geographical region, baseline CD4 + T cell count, and HCV subgenotype. A multivariate logistic regression model was constructed in a forward manner to select variables independently associated with transmission within clusters. Odds ratios (ORs) and adjusted odds ratios (aORs) with 95% confidence intervals (95% CIs) were reported. For all statistical tests, the level of significance for the evaluation of two-sided P values was set at 0.05.

## Results

### Participant characteristics

**Fig. 1:**
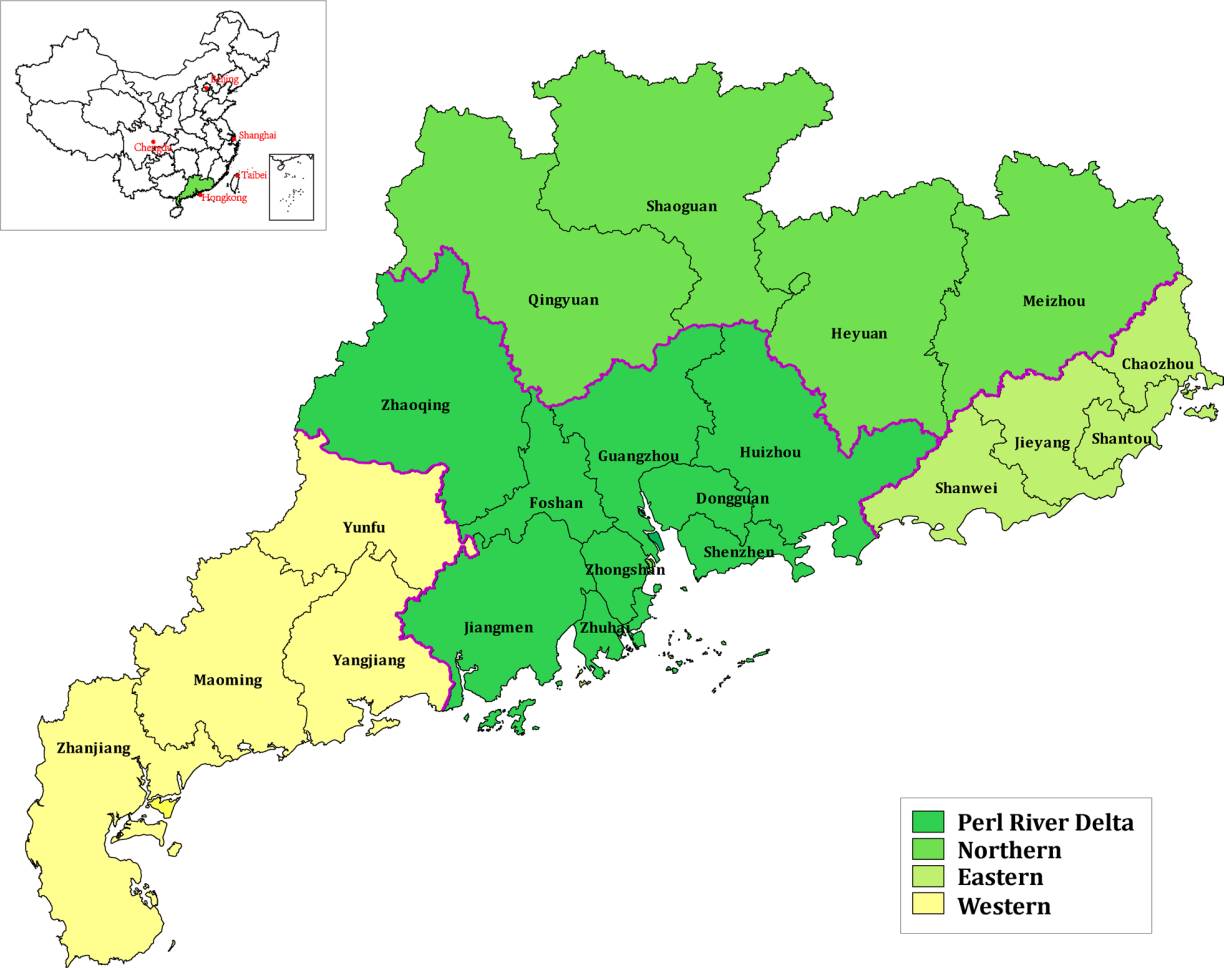
Geographical region of Guangdong province. The geographical regions of Guangdong are represented by different colors on the map. Data is shown on the dataset tabulated in Table 2 and Table 4

For the 356 samples, 302 (84.8%) HCV *NS5B* sequences were successfully amplified, purified, and sequenced. The overall participant characteristics of those with *NS5B* sequences (n = 302) are shown in Table 2. Men constituted 79.47% of the study population. More than 80% of the patients were younger than 50 years when diagnosed. The most common transmission route was injectable drug use (65.89%), followed by heterosexual intercourse (31.46%). More than half of the patients were married or cohabiting (69.54%), and 22.52% were unmarried. The geographical regions of Guangdong mentioned in Table 2 are show in Fig. 1, most patients originated from the Pearl River Delta region (56.62%). A total of 75.83% of the subjects exhibited a baseline CD4^+^ T cell count < 200 cells/mm^3^.

**Table 2:** Characteristics of participants with available HCV *NS5B* segment sequences in HIV/HCV coinfections, 2010–2013, Guangdong, China

Characteristics	Total sequences N = 302 (n/N, %)
*Sex*	
Male	240 (79.47)
Female	62 (20.53)
*Age (years)*	
< 30 years	12 (3.97)
30–39	127 (42.05)
40–49	129 (42.72)
50–59	30 (9.93)
> = 60	3 (0.99)
*Transmission routes*	
Injecting drug use	199 (65.89)
Heterosexual	95 (31.46)
Men who have sex with men	3 (0.99)
Blood	5 (1.66)
*Marital status*	
unmarried	68 (22.52)
Married or cohabiting	210 (69.54)
divorced or separated	16 (5.30)
Widowed	7 (2.32)
unknown	1 (0.33)
*Geographical region*	
Pearl River Delta	171 (56.62)
Eastern	10 (3.31)
Western	101 (33.44)
Northern	20 (6.62)
*Baseline CD*4^+^*T cell count (cells/mm3)*	
< 200	229 (75.83)
200–349	63 (20.86)
350–499	8 (2.65)
> 500	2 (0.66)

### HCV subgenotype determination

A neighbor-joining phylogenetic tree based on the 302 *NS5B* sequences revealed the profile of HCV subgenotypes circulating among HIV/HCV coinfection patients in Guangdong (Fig. 1). Two predominant strains were found to be 6a (58.28%, 176/302) and 1b (18.54%, 56/302), followed by 3a (10.93%, 33/302), 3b (6.95%, 21/302), 1a (3.64%, 11/302), 2a (0.99%, 3/302) and 6n (0.66%, 2/302). There was no significant difference in the distribution of HCV subgenotypes between 2010 and 2013 (Table 3).

**Table 3:** Distribution of HCV subgenotypes in HIV/HCV coinfection patients in Guangdong, stratified by period, 2010–2013 (N = 302)

year	Number(N = 302)	HCV subgenotypes (n/N, %)							P for fisher exact tests
		1a(n = 11)	1b(n = 56)	2a(n = 3)	3a(n = 33)	3b(n = 21)	6a(n = 176)	6n(n = 2)	
2010	117	4 (3.42)	25 (21.37)	1 (0.85)	9 (7.69)	7 (5.98)	70 (59.83)	1 (0.85)	0.951
2011	72	3 (4.17)	12 (16.67)	0 (0.00)	8 (11.11)	7 (9.72)	42 (58.33)	0 (0.00)	
2012	70	2 (2.86)	12 (17.14)	1 (1.43)	10 (14.29)	3 (4.29)	41 (58.57)	1 (1.43)	
2013	43	2 (4.65)	7 (16.28)	1 (2.33)	6 (13.95)	4 (9.30)	23 (53.49)	0 (0.00)	

### Identification of transmission networks

**Fig. 2:**
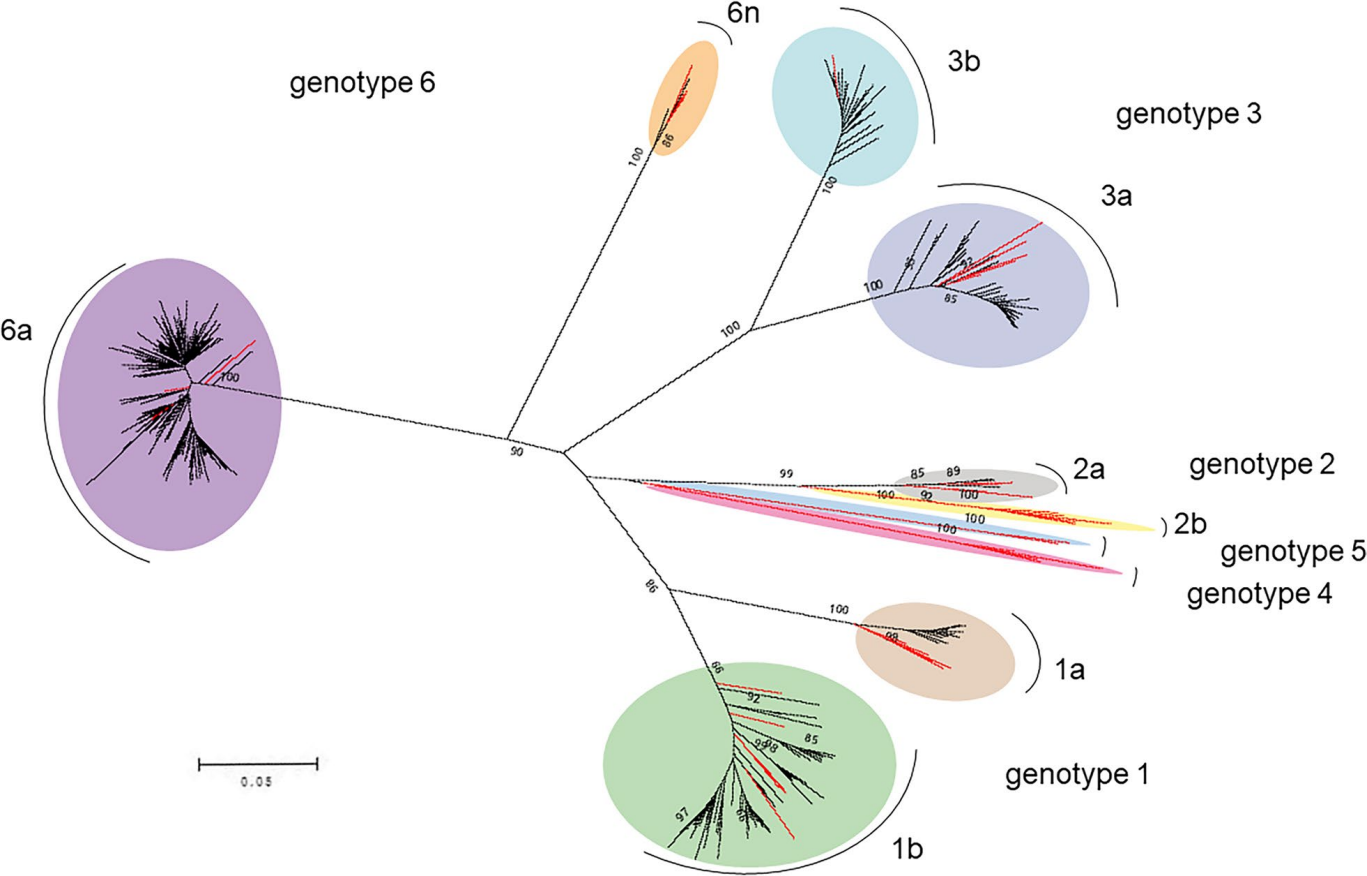
Extensive diversity of HCV subgenotypes among HIV/HCV coinfection patients in Guangdong. A neighbor-joining tree was constructed based on the *NS5B* gene (H77: 7996–8638 nt) of HCV using MEGA 6.0.6 with the Kimura 2-parameter substitution model and 1000 replicates. The black lines represent samples from HIV/HCV coinfection patients in Guangdong, and the red lines represent reference sequences. The bootstrap values related to subtyping are shown in the phylogenetic tree

A total of 11 subgenotype 1a, 56 subgenotype 1b, 33 subgenotype 3a, 21 subgenotype 3b, and 176 subgenotype 6a *NS5B* sequences were used for molecular transmission network analysis between 2010 and 2013. Forty-two transmission clusters containing 133 of the 302 HIV/HCV coinfection patients (total clustering rate: 44.04%) were identified. The average cluster size was 3.24, with a minimum of two (19 clusters) and a maximum of 11 (one cluster). The clustering rates of subgenotypes 1a, 3a, 3b, 1b, and 6a were 18.18% (2/11), 42.42% (14/33), 52.38% (11/21), 48.21% (27/56), and 44.89% (79/176), respectively (Fig. 2).

**Fig. 3:**
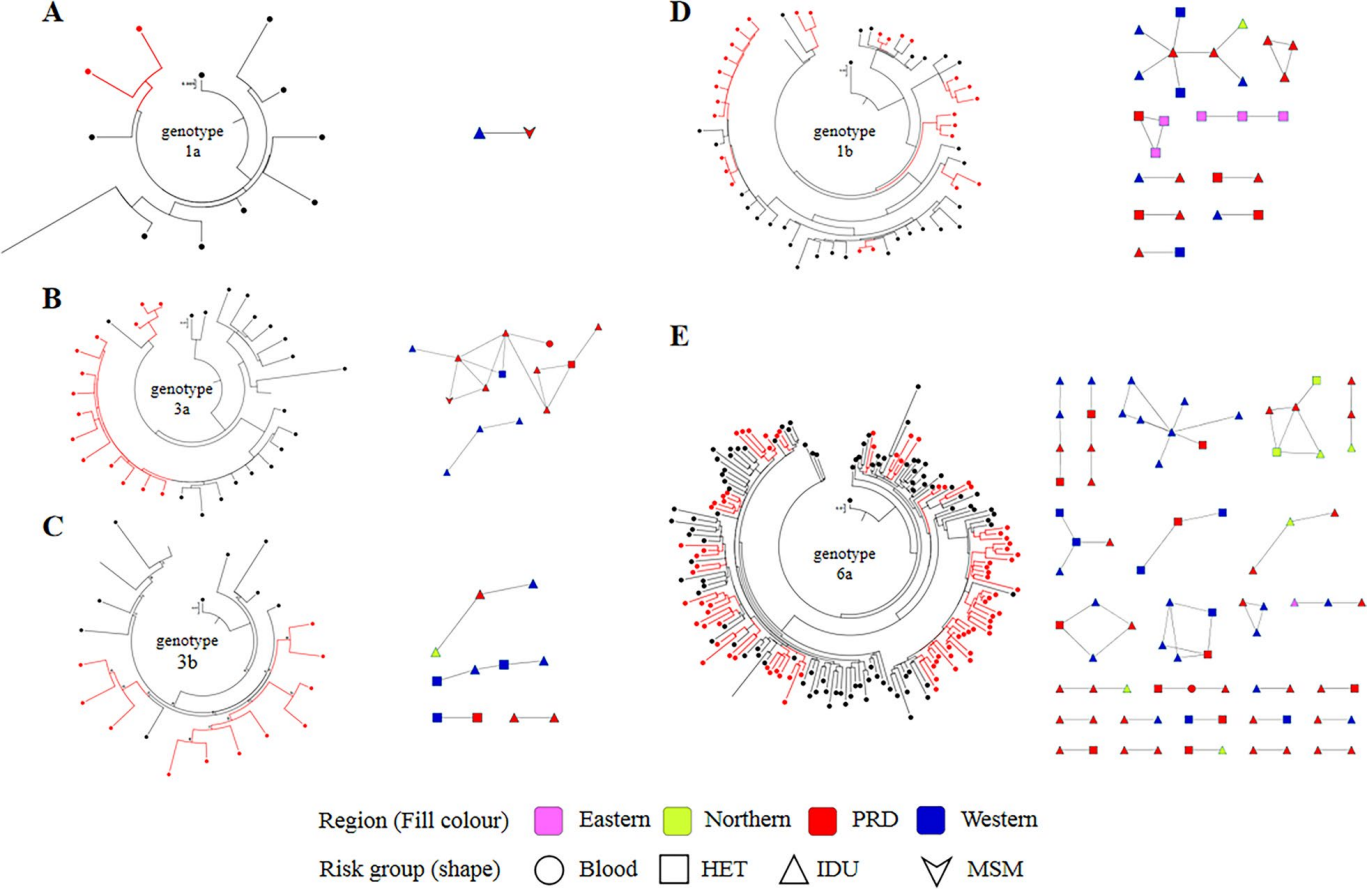
Phylogenetic analysis and transmission networks of genotype 1a- (**A**), genotype 1b- (**B**), genotype 3a- (**C**), genotype 3b- (**D**), and genotype 6a-infected (**E**) individuals. A phylogenetic tree was constructed in PhyML 3.0 using the maximum likelihood method based on the *NS5B* region. The nucleotide substitution model was GTR + G + I. In the circle trees, the red lines with red dots represent samples within clusters, the black lines with black dots represent samples not within clusters, and the black lines without dots represent reference sequences. In the transmission networks, various regions in Guangdong were colour coded. Different shapes represent different risk groups: circle: blood transfusion; square: heterosexual (HET); triangle: injection drug user (IDU); arrowhead: men who have sex with men (MSM)

Among all 42 clusters, 88.10% (37/42) comprised at least one subject from the IDU group, 57.14% (24/42) comprised at least one subject from the HET group, and only 4.76% (2/42) comprised at least one subject from the MSM group or the blood transfusion group (Fig. 3). However, when we analysed the clustering rate of different risk groups, we found that the clustering rate of the MSM group was higher than that of the other groups (66.67% vs. approximately 40%) (Table 4). Of the 133 individuals in clusters, 57.89% (77/133) were linked to cases diagnosed in different regions. Individuals from eastern regions had a higher clustering rate than individuals from other regions (60.00% vs. approximately 40%) (Table 4).

**Table 4:** Factors associated with transmission within clusters

Characteristics	Within transmission network, n = 133 (n/N, %)	Total sequences, N = 302	P for fisher exact tests	OR (95% CI)	P–value	Adjusted OR (95% CI)	P–value
*Sex*							
Male	103 (42.92)	240	0.475	1.000			
Female	30 (48.39)	62		1.247 (0.712–2.183)	0.440		
*Age (years)*							
< 30 years	7 (58.33)	12	0.889	1.000			
30–39	55 (43.31)	127		0.546 (0.164–1.812)	0.322		
40–49	57 (44.19)	129		0.565 (0.170–1.876)	0.351		
50–59	13 (43.33)	30		0.516 (0.134–1.993)	0.337		
> = 60	1 (33.33)	3		0.357 (0.025–5.109)	0.448		
*Transmission routes*							
Injecting drug use	91 (45.73)	199	0.681	1.000			
Heterosexual	38 (40.00)	95		0.791 (0.482–1.300)	0.355		
MSM	2 (66.67)	3		2.374 (0.212–26.603)	0.483		
Blood	2 (40.00)	5		0.791 (0.129–4.839)	0.800		
*Marital status*							
Unmarried	39 (57.35)	68	0.260	1.000		1.000	
Married or cohabiting	84 (40.00)	210		0.496 (0.285–0.863)	0.013	0.496 (0.285–0.863)	0.013
divorced or separated	5 (31.25)	16		0.338 (0.106–1.080)	0.067	0.338 (0.106–1.080)	0.067
Widowed	5 (71.43)	7		1.859 (0.337–10.266)	0.477	1.859 (0.337–10.266)	0.477
unknown	0 (0.00)	1		–	–	–	–
*Geographical region*							
Pearl River Delta	69 (40.35)	171	0.419	1.000			
Eastern	6 (60.00)	10		2.217 (0.603–8.149)	0.230		
Western	49 (48.51)	101		1.393 (0.849–2.287)	0.190		
Northern	9 (45.00)	20		1.209 (0.476–3.073)	0.689		
*Baseline CD*4^+^*T cell count (cells/mm*3*)*							
< 200	104 (45.41)	229	0.852	1.000			
200–349	25 (39.68)	63		0.791 (0.448–1.395)	0.418		
350–499	3 (37.50)	8		0.721 (0.168–3.089)	0.660		
> 500	1 (50.00)	2		1.202 (0.074–19.451)	0.897		
*Subgenotypes*							
1a	2 (18.18)	11	0.282	1.000			
1b	27 (48.21)	56		4.190 (0.830–21.157)	0.083		
2a	0 (0.00)	3		–	–		
3a	14 (42.42)	33		3.316 (0.618–17.800)	0.162		
3b	11 (52.38)	21		4.950 (0.856–28.635)	0.074		
6a	79 (44.89)	176		3.665 (0.770–17.453)	0.103		
6n	0 (0.00)	2		–	–		

Patients were divided according to whether they fell into the transmission networks, and sex, age, transmission route, marital status, geographical region, baseline CD4^+^ T cell count, and subgenotype were examined. The results of the multivariate logistic regression analysis showed that no significant effects from these factors were observed (P > 0.05), except marital status. Married or cohabiting people (compared with unmarried people, aOR = 0.496, 95% CI: 0.285–0.863) had more difficulty forming transmission networks (Table 4).

## Discussion

HCV subgenotypes 1b (62.78%) and 2a (17.39%) were the two predominant subgenotypes in China, according to data from epidemiological studies on hospitalized patients [24]. HCV subgenotypes exhibit significant divergence between regions. HCV subgenotypes 1b and 2a remain the two predominant subgenotypes in North China. While the prevalence of HCV subgenotype 3b in Southwest China is significantly higher than that in other regions [25], HCV 6a was the most frequently represented genotype in southern China [19, 26, 27].

This study revealed that the main circulating HCV subgenotypes among HIV/HCV-coinfected patients in Guangdong were 6a (58.28%, 176/302), followed by 1b (18.54%, 56/302), 3a (10.93%, 33/302), 3b (6.95%, 21/302), 1a (3.64%, 11/302), 2a (0.99%, 3/302), and 6n (0.66%, 2/302). The predominant HCV subgenotypes among HIV/HCV-coinfected individuals in Guangdong were similar to those in Guangxi (6a (46%), 3a (20%), 3b (16%)) [27] but distinct from those in Yunnan (3b (37.62%), 3a (23.76%), 1b (16.34%)) [28]. HCV genotypes vary in the Asia–Pacific region[29], HCV infections and HIV infections have the common transmission route of sharing contaminated injecting equipment, sexual transmission and blood related transmission [29]. The geographic proximity to Southeast Asia and the presence of drug trafficking and use likely explains the similarity of the HCV genotype distributions in HIV/HCV-coinfected individuals between Guangdong and Guangxi. Guangxi Province, which borders Vietnam, could have been the first region to contract 6a for circulation. Genotype 6a was introduced into Guangxi from Vietnam and then further spread to Guangdong through drug trafficking routes and IDU networks [28–30].

The main circulating HCV subgenotypes among HCV mono-infected individuals in Guangdong were 1b (67.7%), followed by 6a (17.2%), 3a (6.1%), 2a (5.0%), 3b (2.0%), 4a (1.0%) and 5a (1.0%) [31],which were quite distinct from that found in the HIV/HCV co-infected patients. The difference in HCV genotype distribution between mono- and co-infection is most likely due to the varied transmission routes, with blood transfusion being the more common route in monoinfection and injectable drug use being the more common route in coinfection [19, 31].

Real-world studies on the efficacy of direct-acting antiviral agents(DAAs) therapy for HCV mono-infected patients in China showed that the sustained virologic response (SVR)12 rate greater than 90% was achieved in most of the HCV genotypes[32, 33]. Subjects with compensated cirrhosis (92.73%) and prior treatment experience (77.78%) had significantly lower SVR rates when compared to chronic hepatitis C (98.15%) and treatment-naive (97.69%) groups[33]. The available DAA regimens were generally well-tolerated and with high efficiency in the treatment of HIV/HCV co-infected patients, with similar efficacy to those with mono HCV infection. There was no significant difference in adverse effects among patients with different baseline CD4^+^ T-cell count in those who received DAA regimens with or without Peg-IFN and RBV[34].

In this study, approximately 44% of the HIV/HCV coinfection patients were members of the HCV transmission networks, which was consistent with the clustering rate of HIV/HCV coinfection patients in Dehong, China [17] (39.1%, 95/243) but higher than the clustering rate of HCV infection patients in Australia (20.76%, 49/236) [9] and Vancouver, Canada (31.14%, 156/501) [35]. Subgenotype 3b and subgenotype 1b inclined to form transmission clusters easily, with comparatively higher clustering rates of 2.38% and 48.21%, respectively. It suggested that the two subgenotypes were transmitted persistently among certain population at high risks, compared to other subgenotypes. According to the results of multivariate logistic regression, sex, age, transmission route, geographical region, baseline CD4 + T cell count and subgenotype were not influencing factors for whether patients entered the transmission networks. Married or cohabiting people had more difficulty forming transmission networks than unmarried people (Table 4), which may be due to the relatively fixed sexual partners of married or cohabiting people, and their probability of high-risk behaviour is lower than that of unmarried people. More than 80% of clusters comprised at least one subject from the IDU group, and in the largest cluster, more than 60% of nodes were patients from the IDU group (Fig. 3). These results suggested that more attention should be given to IDUs in future prevention and control work.

There were several limitations in our study. First, our observations were obtained based on the individuals coinfected with HIV/HCV spanning January 2010 and September 2013 in Guangdong. The shorter terms of recruitment may affected the judgement of HCV prevalence in Guangdong. Second, we focus on the subjects of coinfection which mainly through IDU and heterosexual contact. These specific populations might bias the deduced factors facilitating HCV transmission clustering. Whatever, we indeed performed some work to explore the transmission network of HCV, which may be of help to block the transmission of HCV among HIV individuals and general population.

In conclusion, this study provides an overview of the HCV transmission network among HIV/HCV coinfection patients in Guangdong, China, by using the characteristics of phylogenetic analysis. The total clustering rate was 44.04%, with different subgenotypes varying from 18.18% to 52.38%. Sex, age, transmission route, geographical region, baseline CD4 + T cell count, and subgenotype were not influencing factors, but marital status was an influencing factor for whether subjects entered the transmission network. Additional attention should be given to coinfections among unmarried individuals or patients infected through drug injection in future prevention and control work.

## Data Availability

The datasets used and/or analyzed during the current study are available from the corresponding author on reasonable request.

## Abbreviations

AIDS: Acquired immunodeficiency syndrome
aORs: Adjusted odds ratios
aLRT: Approximate likelihood ratio test
DAA: Direct-acting antiviral agent
HCV: Hepatitis C virus
HIV: Human immunodeficiency virus
HET: Heterosexual
IDU: Injection drug user
MSM: Men who have sex with men
ML tree: Maximum likelihood phylogenetic tree
NS5B: Nonstructural 5B
ORs: Odds ratios
SH: Shimodaira-Hasegawa
SVR: Sustained virologic response
95% CIs: 95% Confidence intervals
